# Clinical characteristics and prognosis of Chinese patients with hereditary transthyretin amyloid cardiomyopathy

**DOI:** 10.1186/s13023-019-1235-x

**Published:** 2019-11-12

**Authors:** Shan He, Zhuang Tian, Hongzhi Guan, Jian Li, Quan Fang, Shuyang Zhang

**Affiliations:** 1 Department of Cardiology, Peking Union Medical College Hospital, Peking Union Medical College and Chinese Academy of Medical Sciences, Beijing, 100005 China; 2 Department of Neurology, Peking Union Medical College Hospital, Peking Union Medical College and Chinese Academy of Medical Sciences, Beijing, 100005 China; 3 Department of Hematology, Peking Union Medical College Hospital, Peking Union Medical College and Chinese Academy of Medical Sciences, Beijing, 100005 China

**Keywords:** Rare disease, Systemic amyloidosis, Transthyretin, Hereditary

## Abstract

**Background:**

Hereditary transthyretin amyloid cardiomyopathy (ATTR-CM) is an increasingly recognized progressive cardiomyopathy with heterogenous clinical manifestations that lead to its misdiagnosis and poor prognosis. This study was performed to describe the clinical characteristics and natural history of Chinese patients to improve clinical awareness of this condition.

**Methods:**

In this study, we retrospectively investigated 23 patients with a confirmed diagnosis of hereditary ATTR-CM in Peking Union Medical College hospital from From January 1, 2000 to December 31, 2018.

**Results:**

In all, 16 patients (69.6%) were males, the median age at disease onset was 45 (33,55) years old. The median duration from symptom onset to diagnosis was 30 (18,46) months. Phenotypes were classified as exclusively cardiac (*n* = 1, 4.3%) and mixed type (*n* = 22, 95.6%). The common mutations were Gly47Arg (7 patients [30.4%]) and Val30Ala (3 patients [13%]). Ventricular hypertrophy was observed in 23 (100%) patients, the mean thickness of the ventricular septum was 16.1 ± 3.9 mm, the mean thickness of the left ventricular posterior wall was 15.1 ± 2.8 mm. The mean left ventricle ejection fraction (LVEF) was 57.3 ± 11.9% and only 5 patients (21.7%) had LVEF < 50%. 18 (78.3%) patients had abnormal electrocardiography and the most common feature was pseudoinfarct pattern (56.5%). Overall survival at 12, 24, 36, 48, and 60 months after diagnosis was 77.8, 55.6, 38.9, 27.8, and 11.1%, respectively. Survival was better in patients with EF ≥50% than in those with EF < 50% [log Rank (Mantel-Cox), χ2 = 4.03, *P* = 0.045].

**Conclusions:**

The clinical characteristics of ATTR are heterogeneous: men are more likely to be affected and onset symptoms are not obvious in the heart and mainly include peripheral neuropathy and autonomic neuropathy; however, LV hypertrophy, especially a thick ventricular septum and posterior wall with preserved LVEF, are often detected on echocardiography. Abnormal ECG manifestations are common. The prognosis is poor, and patients with EF > 50% have better survival. Clinicians should be more aware of the complex clinical profile of ATTR amyloidosis to avoid misdiagnosis in practice.

## Introduction

Amyloidosis is a disordercharacterized by the extracellular deposition of amyloid fibrils in tissues and organs, such as the heart, nerves and eyes [1]. According to the composition of amyloid fibrils, the main amyloidogenic protein-related diseases are identified: immunoglobulin light chain amyloidosis (AL), amyloid A protein amyloidosis (AA), and transthyretin amyloidosis (ATTR) [2]. Transthyretin, a transporter for thyroxine and retinol complex in plasma, is a 55-kD tetrameric protein primarily synthesized in the liver [3, 4]. The TTR gene is located on chromosome 18q12.1 and consists of 4 exons and 5 introns; the 2 distinct types of ATTR are differentiated according to the TTR mutation: hereditary TTR amyloidosis (ATTRm) and wild-type TTR amyloidosis (ATTRwt). Amyloidosis occurs when four monomers dissociated from TTR homotetramers and misfold, aggregate, assemble into amyloid fibrils. The process is facilitated by many factors, including TTR gene mutation, senior age, gender of the transmitting parent and geographical location [5, 6].

Cardiac amyloidosis is partial present in systematic amyloidosis in which the manifestation is irreversible progressive cardiac involvement that eventually develops into fatal restrictive or infiltrative cardiomyopathy, especially in patients with AL amyloidosis or ATTR. ATTR was thought to be much rarer than AL amyloidosis [7]. However, recent studies suggest that transthyretin amyloid cardiomyopathy (ATTR-CM) may be a potentially common condition in the population above 60 years old with heart failure with preserved ejection fraction(HFpEF) [8, 9]. ATTRwt cardiac amyloidosis has been found in 25 to 36% of the population above 80 years old in several autopsy studies [10]. Another autopsy data study demonstrated that TTR amyloidosis occurs in the left ventricular myocardium in 32% of patients with HFpEF who are older than 75 years old [11, 12].

ATTRm also represents a significant portion of ATTR-CM and affects several organs [6]. The clinical spectrum in patients with ATTRm varies widely from exclusive neurological involvement to cardiac involvement diagnosed as cardiomyopathy, supporting the view that heterogeneity in TTR mutations governs the heterogeneity of its phenotypes. Some specific mutations, such as Val122Ile, Thr60Ala, Leu111Met, Ile68Leu, manifest predominantly as cardiomyopathy [13]. The characteristics of cardiomyopathy associated with these specific mutations include restrictive cardiomyopathy with symmetric left ventricular wall and interventricular septum thickening, mild depressed LV ejection fraction values, and no severe symptoms of heart failure after a latent period of autonomic or peripheral neuropathy [14–17]. Specific mutations are associated with geographic distributions and races. African Americans have a high incidence of the TTR mutation Val122Ile, which has an allele frequency of 0.0173 and is carried by 3.5% of African descendants in the United States [18]. Caucasians in Northwest Ireland have a high incidence of the TTR mutation Thr60Ala, where 1% of all Caucasians carry it [19]. Leu111Met and Ile68Leu, are endemic in Denmark and Italy, respectively, and manifest as early onset cardiomyopathy [20, 21].

While the clinical features, heterogeneity of mutations, prognosis have been widely studied in cohorts around the world, clinical studies of ATTRm in Chinese mainland have mainly focused on single mutations limited to single kindred or no more than eight samples [22–25]. The above clinical data focus on the fields of neurology and ophthalmology, but our knowledge of cardiac involvement in this condition is limited. The need for a description of the clinical characteristics of Chinese ATTR-CM is urgent in the challenge of misdiagnosis. Furthermore, several new medications are being explored in clinical trials, such as the ATTR-ACT study, which found that compared to placebo, tafamidis had a significant effect in reducing all-cause mortality and cardiovascular-related hospitalizations [26]. An earlier confirmed diagnosis allows earlier treatment and better prognosis. It is necessary to summarize the profile of hereditary transthyretin amyloid cardiomyopathy to improve clinical awareness and meet the challenge of misdiagnosis. We reviewed 23 patients from unrelated families with a confirmed diagnosis in Peking union medical college hospital (PUMCH) from 2000 to 2018. The aim of this study was to describe the characteristic features associated with hereditary transthyretin amyloid cardiomyopathy in China and analyze its genetic background, disease course, presentation and prognosis.

## Methods

### Design

We performed a retrospective study of all patients with a diagnosis of hereditary ATTR-CM in PUMCH from 2000 to 2018.

### Informed consent

Informed consent was obtained from all participants. All investigations were in accordance with the principles of the Declaration of Helsinki. The study was approved by the ethics committee (EC) of PUMCH.

### Study patients

The inclusion criteria were presentation at the hospitalization with a diagnosis of hereditary ATTR amyloidosis at PUMCH. The diagnosis was verified by clinical symptoms, family history, echocardiography, biopsy and genetic screening. Documentations of the chief complaint, disease course, onset age, family pedigree and race were based on patient self-identification at the initial evaluation. The date of symptom onset was reported by the patient as the date when amyloidosis-associated symptoms first occurred. The date of diagnosis was the date on which the diagnosis of amyloidosis was confirmed histologically. All patients had a positive biopsy showing amyloidosis (endocardium or extracardiac, e.g., abdominal adipose tissue or the gums, rectal mucosa, nerves and muscle), which was defined as apple-green birefringence under polarized light with Congo red staining. All were positive for TTR gene mutations, and no patients with ATTRwt were included. All amyloid patients with elevated light chain levels in the blood/urine/bone biopsy or without confirmation of TTR mutation were excluded. Phenotypes were sorted into (1) Cardiac phenotype: echocardiography (UCG) and/or electrocardiography (ECG) evidence of cardiac amyloidosis in the absence of any sign or symptom of peripheral and/or autonomic neuropathy; (2) Neurologic phenotype: clinical/instrumental evidence of peripheral and/or autonomic neuropathy in the absence of UCG or ECG; (3) Mixed phenotype: cardiac phenotype + neurologic phenotype.

### Diagnostic definitions of hereditary ATTR-CM

Summary of Chinese Hereditary ATTR-CM was defined as echocardiographic findings showing the end-diastolic thickness of the interventricular septum was > 1.2 cm (in the absence of any other plausible causes of LV hypertrophy) and/or ECG low voltage (QRS amplitude ≤0.5 mV in all limb leads or < 1 mV in all precordial leads) advanced A-V block or intraventricular conduction disturbances in patients with ATTR. Other echocardiographic signs suggesting cardiac amyloidosis (in addition to increased LV wall thickness) were systematically checked and included granular sparkling appearance of the ventricular myocardium, increased thickness of the atrioventricular valves or interatrial septum, pericardial effusion [15].

### Genotyping

Genomic DNA was isolated from whole peripheral blood by standard techniques. Exons 2, 3, and 4 of the TTR gene (accession number m11844) were amplified by polymerase chain reaction (Takara ExTaq polymerase) using previously described primers. In all, 13 amplified DNA fragments were directly sequenced using an ABI Prism 3130 automated sequencer.

### Survival analyses and statistics

Summary statistics are expressed as the median (interquartile range) or numbers (percentages). The independence of continuous variables was tested using the Mann-Whitney U test/Kruskal-Wallis test. Kaplan-Meier survival was calculated from the date of the original diagnosis to the date of death or the most recent contact. Mortality information were obtained for follow-up analyses by regular telephone contact. Because this study was retrospective, clinical documentation and mortality information were used to ensure comprehensive follow-up information. All tests were 2-tailed, *P* value of < 0.05 was considered statistically significant. Statistical analyses were performed using SPSS Statistics (version 22. IBM Corp., Chicago, IL, USA).

## Results

### Patients

The 23 Chinese patients with hereditary cardiac transthyretin amyloidosis were referred from many different clinical specialties: 5 were diagnosed by cardiologists at the first visit, 11 by neurologists, and 7 by gastroenterologists. Twenty-one patients were ethnic Han-Chinese, and the remaining two were ethnic Hui-Chinese or ethnic Mongolian-Chinese. Only 9 cases (39.1%) had a defined family history of amyloidosis.

### DNA analysis

DNA sequencing of the TTR gene demonstrated that all 23 patients were heterozygous for previously reported mutations. A total of 15 kinds of TTR mutations were identified, and the most common mutations were Gly47Arg (7 patients [30.4%]) and Val30Ala (3 patients [13%]). Other less common mutations were found in 1 patient each (His88Arg, Tyr69His, Glu61Lys, Ser77Tyr, Val30Met, Phe33Leu, Gly53Glu, Gly47Glu, Tyr114Cys, Glu54Lys, Asp38Val, Lys35Asn, and Glu54Gln) (Table1).

### Histology

Congo red staining of biopsies was performed in all patients. Biopsies of the peripheral nerve, abdominal fat, gingiva and tongue were performed in all patients, but biopsies of the endocardium (5/7), thyroid (1/7) and brain (1/7) were performed in only 7 patients. Five patients underwent TTR immunohistochemistry in the peripheral nerve. Positivity was found in the following sites: the gingiva (9/23, 39.1%), heart (5/5, 100%), abdominal fat (12/23, 52.2%), peripheral nerve (18/23, 78.3%), brain (1/1, 100%), and thyroid (1/1, 100%), and TTR immunohistochemistry was performed in the peripheral nerve (5/5, 100%) (Table 1).

**Table 1:** Summary of Chinese patients with hereditary transthyretin cardiac amyloidosis

Patient No.	Sex	Age at onset (years)	Age at diagnosis (years)	Mutation	Initial manifestation	Cardiac abnormalities	Phenotype	Positive tissue biopsy
1	M	33	34	Gly47Arg	Paresthesia	CA, HF, A	Mixed	G H AF PN
2	M	49	50	Gly47Glu	Orthostatic hypotension	CA, HF, A	Mixed	PN
3	F	42	45	Val30Ala	Paresthesia	CA, A	Mixed	T AF PN
4	F	62	64	His88Arg	Dyspnea	CA, HF, A	cardiac	G H PN
5	M	27	30	Val30Ala	Paresthesia	CA, A	Mixed	AF PN
6	F	34	39	Gly53Glu	Impaired consciousness, paresthesia	CA, A	Mixed	AF PN B
7	M	30	34	Gly47Arg	Orthostatic hypotension	CA, A	Mixed	G AF PN
8	M	61	65	Tyr69His	Impaired consciousness	CA	Mixed	B
9	M	37	39	Glu54Gln	Paresthesia, diarrhea	CA,	Mixed	PN
10	M	17	19	Glu47Arg	Impaired consciousness, paresthesia	CA, A	Mixed	AF PN
11	F	25	27	Glu47Arg	Orthostatic hypotension	CA, A	Mixed	PN
12	M	53	57	Val30Met	Paresthesia	CA	Mixed	PN
13	M	55	61	Glu47Arg	Paresthesia	CA, A	Mixed	G H AF PN
14	M	45	47	Glu54Gln	Paresthesia, diarrhea	CA	Mixed	PN
15	M	60	68	Glu61Lys	Paresthesia, diarrhea	CA,	Mixed	G H AF PN
16	M	30	32	Gly47Arg	Paresthesia	CA,	Mixed	G
17	F	35	38	Gly47Arg	Paresthesia	CA,	Mixed	G AF PN TH
18	M	49	52	Asp38Val	Orthostatic hypotension	CA, HF, A	Mixed	H AF PN
19	M	50	52	Phe33Leu	Paresthesia	CA, HF	Mixed	PN
20	F	48	50	Lys35Asn	Diarrhea	CA,	Mixed	PN
21	M	29	31	Tyr114Cys	Paresthesia, vomit	CA, A	Mixed	G
22	F	58	60	Val30Ala	Orthostatic hypotension	CA, A	Mixed	G AF PN
23	M	56	60	Ser77Tyr	Diarrhea	CA, A	Mixed	G AF

*A* abnormal ECG, *CA* cardiac amyloidosis, *F* female, *HF* heart failure, *M* male, *G* gingiva, *T* tongue, *H* heart, *AF* abdominal fat, *C* cataract, *PN* peripheral nerve, *B* brain, *TH* thyroid

### Clinical presentation and course

The median (Q1,Q3) age at onset was 45 (33,55) years old, at diagnosis was 47 (34,60) years old. The median time from symptom onset to diagnosis was 30 (18,46) months. According to onset age whether above 50 years old, 8 patients were in the late-onset group and there was no common mutation among these 8 patients (mutations in the late-onset group: His88Arg, Tyr69His, Glu61Lys, Val30Ala, Ser77Tyr, Glu47Arg, Val30Met, and Phe33Leu). Fifteen patients were in the early-onset group (mutations in the early-onset group: Glu47Arg, Val30Ala, Gly47Glu, Tyr114Cys, Glu54Lys, Asp38Val, Lys35Asn, and Glu54Gln). There were 2 common genotypes (Glu47Arg and Val30Ala), which were found in 6 and 2 early-onset probands, respectively. Clinical presentations involved the heart, peripheral nerve, autonomic nerve and central nerve. Cardiac presentations were found in 5/23 (21.7%) patients (No. 1, 2, 4, 18, and 19). Autonomic and peripheral nerve dysfunction were found in 21/23 (91.3%) and 22/23 (95.7%) patients, respectively, and developed concomitantly in 21 cases (Table 2). Although only 21.7% of the patients presented with cardiac symptoms, 23/23 (100%) had echocardiographic evidence of cardiac amyloidosis, and 14/23 (61%) had ECG abnormalities at diagnosis (Table 3).

**Table 2:** Neurological features at diagnosis

	*N* = 23 (%)
Autonomic neuropathy	21 (91.3)
Orthostatic hypotension	5 (21.7)
Constipation	2 (8.7)
Diarrhea	8 (34.8)
Upper gastrointestinal tract symptoms (early satiety, dyspepsia, dysphagia, vomiting)	5 (21.7)
Weight loss	7 (30.4)
Urinary retention	2 (8.7)
Impotence	4 (17.4)
Peripheral neuropathy	22 (95.7)
No peripheral or autonomic neuropathy	1 (4.3)

**Table 3:** Echocardiography and electrocardiogram at diagnosis

	N = 23 (%)
Echocardiography	
LVH absent other causes	23 (100.0%)
LVH pattern:	
1. Symmetric	22 (95.6%)
2. Asymmetric	1 (4.3%)
Systolic dysfunction	5 (21.7%)
Diastolic dysfunction	23 (100.0%)
Restrictive filling	9 (39.1%)
Pericardial effusion	10 (43.5%)
Granular sparkling appearance	18 (78.3%)
Left atrial dilation	5 (21.7%)
LVEF < 50%	5 (21.7%)
LVH + pericardial effusion	10 (43.5%)
Electrocardiogram	
Normal ECG	5 (21.7%)
LV hypertrophy on ECG	2 (8.7%)
Low voltage limb/precordial leads	8 (34.8%)
Pseudoinfarct pattern	13 (56.5%)
AV block	3 (13.1%)
Pacemaker	2 (8.7%)
Atrial fibrillation	1 (4.3%)
RBBB	2 (8.7%)
LBBB	1 (4.3%)
Echocardiography + electrocardiogram	
LVH + low/ normal QRS voltage	9 (73.3%)

*AV* atrioventricular, *L/RBBB* left/right bundle branch block, *LVH* left ventricular hypertrophy

### Echocardiographic characteristics

Increased LV wall thickness and diastolic dysfunction were observed at diagnosis in 23 patients (100%). The pattern of LV hypertrophy was symmetric in 22 individuals (95.6%) and asymmetric in 1 patient (4.3%). A granular sparkling appearance was found in 18 patients (78.3%). Five patients (21.7%) had systolic dysfunction with ejection fraction <50%. Pericardial effusion was present in 10 patients (43.5%). Nine patients (39.1%) showed a restrictive filling pattern. Five patients (21.7%) had left atrial dilation (Table 3). The echocardiographic parameters at diagnosis are outlined in Table 4. The mean LVEF was 57.3 ± 11.9%, and 5 patients (21.7%) had a LVEF < 50%. The mean Interventricular septal thickness was 16.1 ± 3.9 mm, and the mean left atrial diameter was 34.3 ± 5.9 mm (Table 4).

**Table 4:** Echocardiography at diagnosis

Echocardiography	Values	Normal values	Percentage abnormal (%)
Left ventrium			
Interventricular septal thickness (IVS) (mm)	16.1 ± 3.9	7–11	100
Left ventricular posterior wall thickness (LVPW) (mm)	15.1 ± 2.8	7–11	100
left ventricular internal dimension in systole(LVIDs)(mm)	33.9 ± 10.7	35–55	4.3
left ventricular internal dimension in diastole(LVIDd)(mm)	48.5 ± 6.3	25–40	0
Left ventricular ejection fraction (LVEF) (%)	57.3 ± 11.9	50–75	17.4
Left atrium			
Left atrial diameter (LAd) (mm)	34.3 ± 5.9	19–39	21.7
Left atrial area (cm2)	21 ± 4.3	< 20	74.1

### Electrocardiogram characteristics

Electrocardiogram abnormalities were evident in 18/23 (78.3%) cases at diagnosis. 8/23 (34.8%) patients had low QRS voltage according to limb and precordial leads criteria (Table 3). LV hypertrophy on ECG was present in 2/23 (8.7%) patients. However, a pseudoinfarct pattern is common, observed in precordial leads in 13/23 (56.5%) patients (Table 3). AV block was found in 2/23 (8.7%) patients who were later equipped with permanent pacemakers. Only 1/23 (4.3%) patient suffered from atrial fibrillation (Table 3).

### Survival

**Fig. 1:**
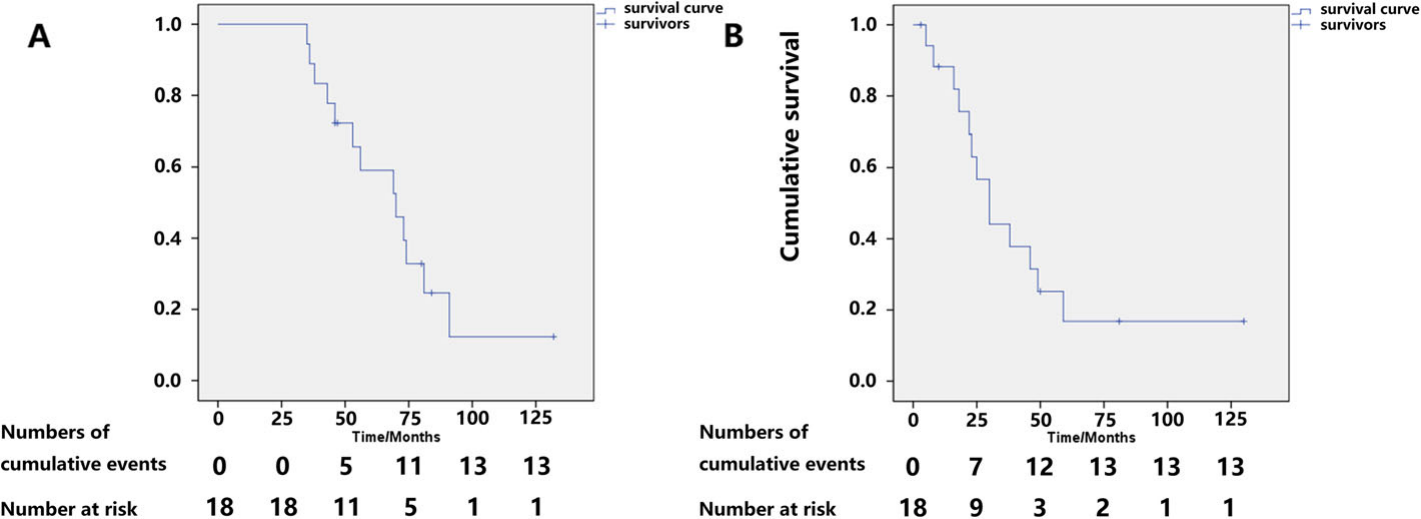
(**a**) Kaplan-Meier survival curves of data obtained from symptom onset. The date of symptom onset was reported by the patient as the date when amyloidosis-associated symptoms first occurred. Overall survival at 12, 24, 36, 48, and 60 months after onset was 100.0, 100.0, 88.9, 61.1, and 50.0%, respectively. Numbers below the plot represent number of subjects at risk and at each time point. Cumulative events mean all-cause death in subjects. Total mortality was 72.2% during a median follow-up of 62.5 (45.25,80.25) months. The median survival time from onset was 70.0 (95CI: 48.9–91.1) months. (**b**) Kaplan-Meier survival curves at confirmed diagnosis. The date of diagnosis was the date on which the diagnosis of amyloidosis was confirmed histologically. Overall survival at 12, 24, 36, 48, and 60 months after diagnosis was 77.8, 55.6, 38.9, 27.8, and 11.1%. Numbers below the plot represent number of subjects at risk and at each time point. Cumulative events means all-cause death in subjects. The median survival time from diagnosis was 30.0 (95CI: 20.3–39.7) months

**Fig. 2:**
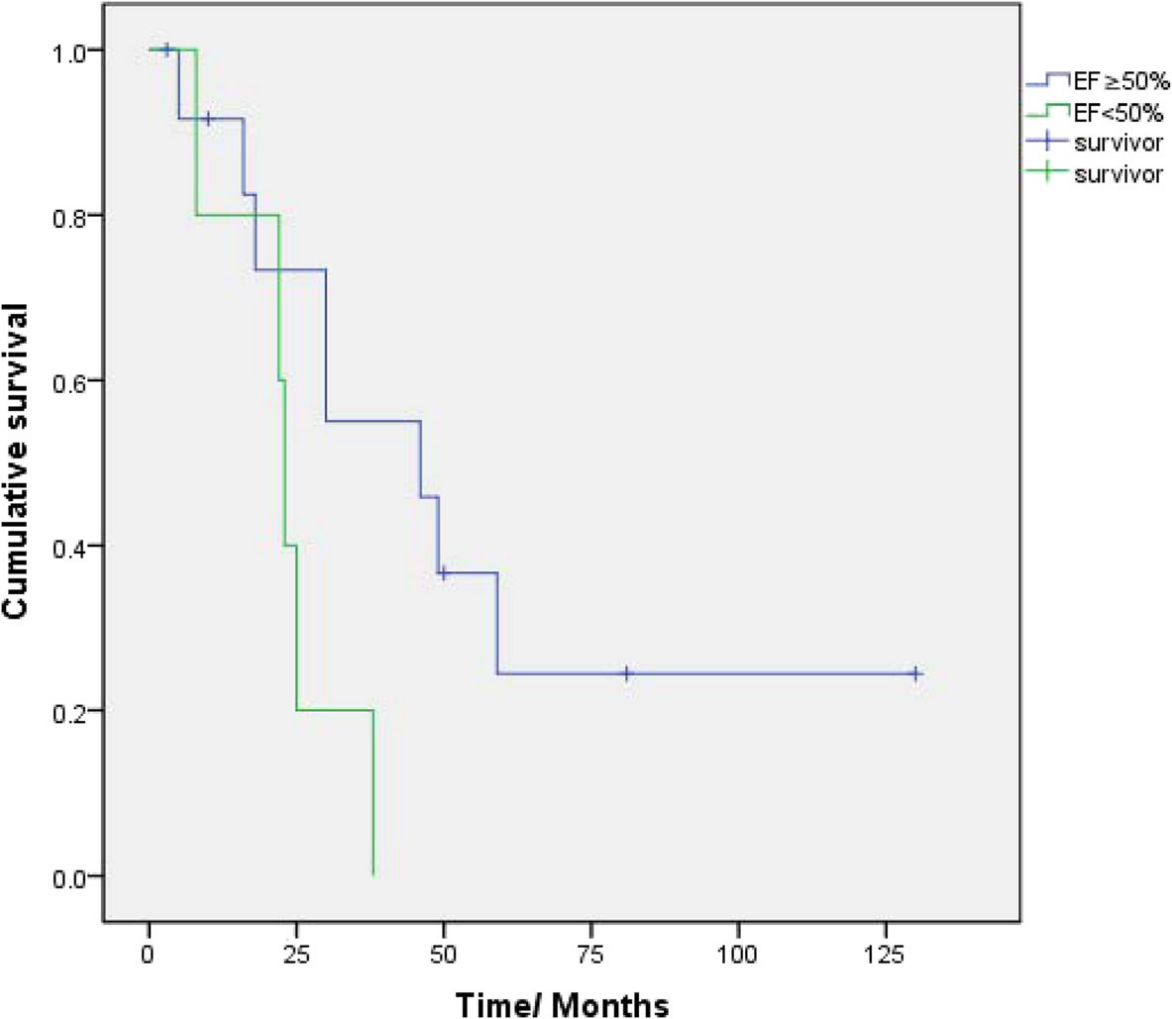
Kaplan-Meier survival curves for patients grouped by EF% at diagnosis. Survival was better in patients with EF ≥ 50% than in those with EF < 50% at diagnosis [log Rank (Mantel-Cox), χ2 = 4.03, P = 0.045]. The median survival time of EF ≥ 50% was 46.0 (95CI: 15.5–76.5) months. The median survival time of EF < 50% was 23.0 (95CI: 20.8–25.1) months. HFrEF indicates the poor prognosis in ATTR-CM

We summarized survival results in 18/23 patients (5 patients were lost to follow-up). The median survival times after onset and diagnosis were 70.0 (95CI: 48.9–91.1) months and 30.0 (95CI: 48.9–91.1) months, respectively. A total of 13 patients died. The mortality rate was 72.2%. Deaths resulted from heart failure (*n* = 7), sudden death (*n* = 3), sepsis after liver transplantation (*n* = 1), and unclassified (*n* = 2). The median time from onset to diagnosis (delay of diagnosis) was 30 (18,46) months. Overall survival after onset at 12, 24, 36, 48, and 60 months was 100.0, 100.0, 88.9, 61.1, and 50.0%. Overall survival after diagnosis at 12, 24, 36, 48, and 60 months was 77.8, 55.6, 38.9, 27.8, and 11.1% (Fig. 1). There was evidence showing that survival was better in patients with EF ≥ 50% than in those with EF < 50% [log Rank (Mantel-Cox), χ2 = 4.03, *P* = 0.045] (Fig. 2).

## Discussion

More than 120 TTR mutations have been widely reported around the world, with affected patients showing varied clinical presentation and disease progression. ATTR is classified into three main phenotypes (neurological, cardiac, and mixed), and its cardiac phenotypes is defined as hereditary ATTR-CM [27]. Emerging data suggest that ATTR-CM will become the most commonly diagnosed form of cardiac amyloidosis [15, 28]. In this study, we describe the clinical features, natural history and outcomes of hereditary ATTR-CA based on 18 years of experience at a large single-center institution. This is the largest study performed in mainland China to date.

Although autosomal dominant inherited diseases generally occur equally between males and females, we noted a male predominance of this condition of nearly 2:1. This male preference has also been reported in other countries, but the underlying mechanism is unknown. A defined family history was found in only 39.1% of the probands from different families, and we therefore could not roughly exclude suspected patients without a defined family history. The lack of identifiable family history and the sexual predominance of this condition may be explained by variable penetrance of the mutant allele [29].

The heterogeneity of these mutations is evident. A total of 15 kinds of TTR mutations were identified in 23 probands from different families. The common mutations were Gly47Arg (7 patients [30.4%]) and Val30Ala (3 patients [13%]). However, two widely reported mutations (Val122Ile and Val30 Met) and other mutations known to prevalent in western world (Thr60Ala, Leu111 Met, and Ile68Leu) were not found in our Chinese population. This finding supports the notion that the heterogeneity observed in mutations is related to race and geography. The 2 common mutations occurred in different races within China. Gly47Arg was observed in ethnic Han-Chinese and ethnic Hui-Chinese, whereas Val30Ala was observed in ethnic Han-Chinese and ethnic Mongolian-Chinese. Therefore, China contains a gene pool with potentially highly heterogeneous TTR mutations.

The heterogeneity of the manifestations among affected individuals is evident. Although all patients consistently obtained a diagnosis of hereditary cardiac transthyretin amyloid cardiomyopathy, their initial manifestations were usually peripheral neuropathy (95.7%) and autonomic neuropathy (91.3%), which developed concomitantly in 21 cases. Peripheral neuropathy always presented as sensorimotor neuropathy, while the presentation of autonomic neuropathy was more complex. Diarrhea (34.8%), orthostatic hypotension (21.7%) and upper gastrointestinal tract symptoms (early satiety, dyspepsia, dysphagia, and vomiting) (21.7%) were the main manifestations of autonomic neuropathy. We categorized diarrhea and upper gastrointestinal tract symptoms as autonomic neuropathy because the above symptom-related patients all had negative results for Congo-red staining in rectum biopsies. However, the exclusively neurological type was absent, the purely cardiac type (patient No. 4 patient with a His88Arg mutation) accounted for only 4.3% of the patients, and the mixed type (neurological + cardiac) was the most common type in this study (91.3%). This result is accordance with the spectrum of genotype-phenotype correlations reported in Italian cohorts, in which ATTR varied widely from exclusively neurological involvement to exclusively cardiac involvement. In between these two extremes, patients with several TTR mutations, presented with variable degrees of neurological and cardiological involvement [15].

Electrocardiographic abnormalities were evident in 18/23 (78.3%) patients. Although low-voltage QRS is widely considered the most common ECG sign of cardiac amyloidosis [30], we found that this feature was only found in 8/23 (34.8%) patients. This finding supports the notion that low-voltage QRS is more highly prevalent in AL amyloidosis than in ATTR [31]. A pseudoinfarct pattern (mainly in anterior and lateral leads) was the most common ECG finding and was observed in 13/23 (56.5%) patients. Amyloidosis may also infiltrate the cardiac conduction system and cause different types of heart arrhythmias. Advanced AV block was found in 2/23 (8.7%) patients, and these patients were finally equipped with permanent pacemakers. Only 1/23 (4.3%) patient suffered from atrial fibrillation. Despite the low sensitivity and specificity of ECG, it should be performed in suspected patients because advanced AV block is an independent factor associated with sudden death in patients with cardiac amyloidosis [32]. Because a normal ECG does not exclude cardiac amyloidosis, echocardiography is recommended.

The hallmark echocardiography result in these patients is symmetric LV hypertrophy (LVH) and diastolic dysfunction coexistent with pericardial effusion. LVH was observed at diagnosis in all patients. The pattern of LVH was symmetric in 22 individuals (95.6%) and asymmetric in 1 patient (4.3%). A granular sparkling appearance was found in 18 patients (78.3%). Five patients (21.7%) had systolic dysfunction with ejection fraction <50%. Pericardial effusion was present in 10 patients (43.5%). Nine patients (39.1%) showed a restrictive filling pattern. Five patients (21.7%) had left atrial dilation. Mean LVEF was 57.3 ± 11.9%, and only 5 patients (21.7%) had LVEF < 50%. The above findings have also been observed in studies of ATTR-CM, and the hallmark features of echocardiography associated with this disease may provide high sensitivity for cardiac amyloidosis and support its differential diagnosis with HCM.

The prognosis of hereditary cardiac transthyretin amyloidosis is not optimistic. In this study, total mortality was 72.2% during a median follow-up of 62.5 (45.25,80.25) months. The median survival times from onset and diagnosis were 70.0 (95CI: 48.9–91.1) months and 30.0 (95CI: 20.3–39.7) months, respectively. Deaths resulted from heart failure (*n* = 7), sudden death (*n* = 3), sepsis (*n* = 1), and unclassified (*n* = 2). Overall survival at 12, 24, 36, 48, and 60 months after onset was 100.0, 100.0, 88.9, 61.1, and 50.0%, respectively. Overall survival at 12, 24, 36, 48, and 60 months after diagnosis was 77.8, 55.6, 38.9, 27.8, and 11.1%, respectively (Fig. 1). Survival was better in patients with EF ≥ 50% than in those with EF < 50% [log Rank (Mantel-Cox), χ2 = 4.03, *P* = 0.045]. The results obtained for EF were statistically significant based on the limited sample size, which indicates that reduced EF at confirm diagnosis is associated with a poor prognosis and indicates HFpEF progress into HFrEF.

A poor prognosis may be associated with several factors. First, a delay of diagnosis. The median time from onset to diagnosis (delay of diagnosis) was 30 (18,46) months and the longest time was 61 months. Second, the features indicating delayed dominance and incomplete penetrance in this autosomal dominant diseases may contribute to a delay in diagnosis even among patients with a defined family history. Third, the need for a histological evidence of target organ amyloid infiltration can also delay the diagnosis because abdominal fat biopsy has limited value in diagnosing this condition and the use of TTR pathologic analysis, endomyocardial biopsy, DNA techniques, and nuclear medicine are restricted to referral or advanced centers. Therefore, clinical awareness needs to be improved. An early diagnosis not only allows the identification of patients with hereditary ATTR amyloidosis before the onset of multiorgan failure but also magnifies the benefits of organ transplantation or the use of TTR stabilizers and mRNA interference.

### Limitations

This study has several weaknesses. First, this was a retrospective study that included limited patients, and 5 of them were lost to follow-up. Second, not all patients were diagnosed during the early stage of the disease, and the data for these patients may not correctly reflect the actual genotype-phenotype relationships. Third, there was relatively conclusive evidence showing that survival was better in patients with EF ≥ 50% than in those with EF < 50% [log Rank (Mantel-Cox), χ2 = 4.03, *P* = 0.045]; however, this result was limited by the size of our samples and the existence of lost follow-up. Finally, nuclear scintigraphy is the current golden standard but it was not applied in all patients. Further studies will be needed to investigate the early-stage clinical manifestations of TTR mutation types in mainland China and expand the size of the sample population. Family pedigrees with the Gly47Arg and Val30Ala mutations should be investigated to profile the natural history of this condition and identify the differences between asymptomatic carriers and patients.

## Conclusion

Clinical characteristics of hereditary ATTR-CM are heterogeneous: Men are more likely to be affected, and its onset symptoms are not obvious in the heart but are instead mainly linked to peripheral neuropathy and autonomic neuropathy. However, LV hypertrophy, especially a thick ventricular septum and posterior wall, combined with preserved LVEF were detected on echocardiography. Abnormal ECG manifestations were common. The prognosis was poor, and survival was better in patients with ≥50%. Clinicians are aware of the complex clinical profile of ATTR amyloidosis to avoid misdiagnosis in practice.

## Data Availability

The datasets used and/or analysed during the current study are available from the corresponding author on reasonable request.

## Abbreviations

ATTR: Transthyretin amyloidosis
ATTR-CM: Hereditary transthyretin amyloid cardiomyopathy
ATTRm: Hereditary TTR amyloidosis
ATTRwt: Wild-type TTR amyloidosis
LVEF: Left ventricle ejection fraction
TTR: Transthyretin
